# Florid inflammatory reaction after fat grafting into an irradiated DIEP-reconstructed breast mimicking cancer recurrence

**DOI:** 10.1093/jscr/rjag597

**Published:** 2026-07-16

**Authors:** Manasseh Tamakloe, Sarah Omar, Krzysztof Sosnowski, Simon Russell, Parto Forouhi, Charles M Malata

**Affiliations:** Plastic Surgery Department, Nuffield Health Cambridge Hospital, 4 Trumpington Rd, Cambridge CB2 8AF, United Kingdom; Plastic Surgery Department, Addenbrooke’s Hospital, Cambridge University Hospitals NHS Foundation Trust, Hills Rd, Cambridge CB2 0QQ, United Kingdom; School of Clinical Medicine, University of Cambridge, Hills Rd, Cambridge CB20SP, United Kingdom; Plastic Surgery Department, Nuffield Health Cambridge Hospital, 4 Trumpington Rd, Cambridge CB2 8AF, United Kingdom; Department of Radiotherapy, Addenbrooke’s Hospital, Cambridge University Hospitals NHS Foundation Trust, Hills Rd, Cambridge CB2 0QQ, United Kingdom; Cambridge Breast Unit, Addenbrooke’s Hospital, Cambridge University Hospitals NHS Foundation Trust, Hills Rd, Cambridge CB2 0QQ, United Kingdom; Plastic Surgery Department, Nuffield Health Cambridge Hospital, 4 Trumpington Rd, Cambridge CB2 8AF, United Kingdom; Cambridge Breast Unit, Addenbrooke’s Hospital, Cambridge University Hospitals NHS Foundation Trust, Hills Rd, Cambridge CB2 0QQ, United Kingdom; Plastic Surgery Department, Nuffield Health Cambridge Hospital, 4 Trumpington Rd, Cambridge CB2 8AF, United Kingdom; Plastic Surgery Department, Addenbrooke’s Hospital, Cambridge University Hospitals NHS Foundation Trust, Hills Rd, Cambridge CB2 0QQ, United Kingdom; Cambridge Breast Unit, Addenbrooke’s Hospital, Cambridge University Hospitals NHS Foundation Trust, Hills Rd, Cambridge CB2 0QQ, United Kingdom; Anglia Ruskin University School of Medicine, Anglia Ruskin University, East Rd, Cambridge CB1 1PT, United Kingdom

**Keywords:** fat grafting, breast reconstruction, fat necrosis, radiotherapy, fat grafting complications, autologous fat transfer

## Abstract

Autologous fat grafting (AFG) is widely used to refine contour and volume following breast reconstruction. However, its use in previously irradiated fields carries a higher risk of complications and poses diagnostic challenges in excluding locoregional recurrence. We report an unusual early inflammatory reaction following AFG into an irradiated Deep Inferior Epigastric Perforator flap-reconstructed breast, characterized by florid indurated, erythematous nodules within the fat-grafted native chest wall skin that clinically mimicked cancer recurrence. The lesions resolved with conservative management, and the patient uneventfully underwent further stages of lipofilling for symmetry. We postulate that the reaction represents an exaggerated inflammatory response to injected fat within a radiation-damaged microenvironment, a phenomenon not previously described in native irradiated breast skin following AFG. This case highlights an unreported complication of AFG and the diagnostic challenges it may pose. Clinicians should maintain a high index of suspicion and biopsy and image any suspicious lesion in these patients.

## Introduction

Fat grafting is widely used for the correction of contour irregularities and volume deficits in postmastectomy and postlumpectomy breast reconstruction. It utilizes autologous fat harvested from other anatomical sites, prepared and transferred to the breast either before, during, or after breast reconstruction [1, 2]. Besides improving aesthetic outcomes, its oncological safety is supported by several studies, with none demonstrating increased loco-regional recurrence risk [3]. Its short-term complications include infection, fat necrosis, oil cysts, calcifications, and palpable breast nodules, which frequently necessitate further investigations [4].

Postmastectomy radiotherapy (PMRT) reduces local relapse and improves survival, but its complications diminish quality of life and breast reconstruction outcomes, even with autologous techniques [5], [6] Fat grafting offers a low-morbidity option for improving soft-tissue contour and the quality of radiation-damaged skin, but the irradiated bed itself alters the biological response to the injected graft. The behaviour of fat in irradiated native skin remains incompletely characterized, and complications in this setting are infrequently reported.

The patient herein reported presented with an atypical tissue reaction following fat grafting into an irradiated Deep Inferior Epigastric Perforator (DIEP) flap-reconstructed breast mound. This presentation raised suspicion of local tumour recurrence and prompted additional evaluation.

## Case presentation

A 57-year-old female presented with bilateral invasive grade 2 lobular carcinomas (Estrogen Receptor/Progesterone Receptor positive, Human Epidermal Growth Factor Receptor 2 negative), node-negative, right 24 mm, left 70 mm. Her medical history included hypertension and previous Crohn’s disease; she was a non-smoker and teetotaler. She underwent primary bilateral skin-sparing mastectomies with sentinel lymph node biopsy and immediate DIEP flap reconstruction; recovery was uneventful.

**Figure 1:**
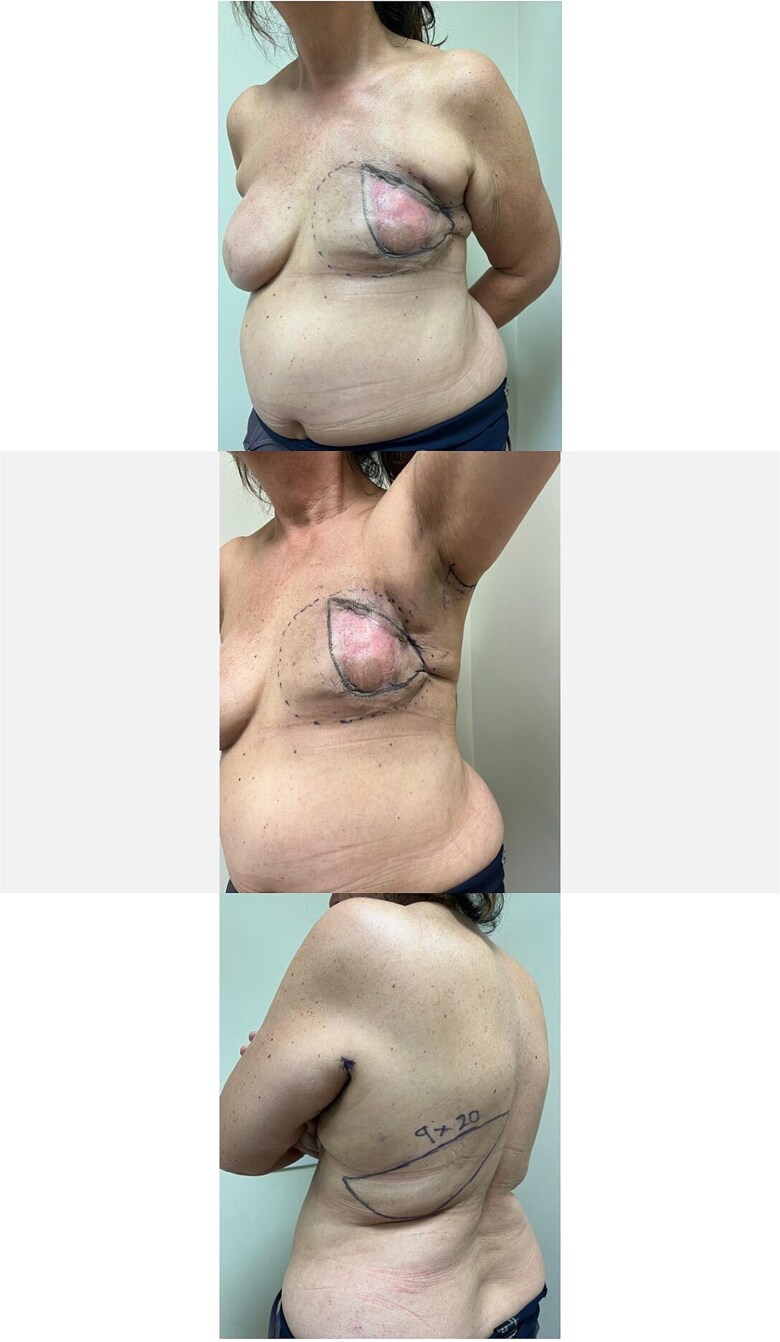
Effects postradiotherapy: pocket shrinkage, fat necrosis, contracture and loss of volume, skin grafted area laterally necessitating salvage reconstruction with an LD flap; the surgical planning is demonstrated.

At 3 months, she received left-breast PMRT 26 Gy in 5 fractions. This was complicated by wound breakdown and chronic sinus formation, requiring multiple debridements of the necrosed areas and split-thickness skin grafting. Persistent fat necrosis and discharging sinuses necessitated salvage reconstruction with a latissimus dorsi (LD) myocutaneous flap (Fig. 1). Six months post mastectomy, volume and contour deficits with attendant poor surrounding soft tissue cover prompted fat grafting (Lipografter **®** technique) of 270 ml of fat into the irradiated chest wall skin (surrounding the LD flap skin paddle) using fat from the abdominal dog-ears and the mons pubis.

**Figure 2:**
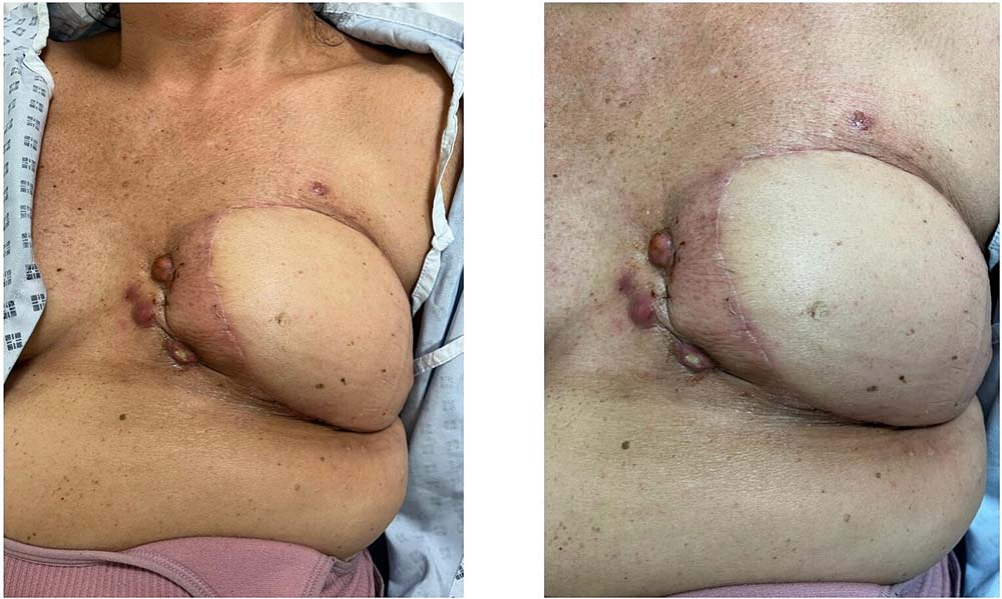
Florid tissue reaction of nodules 3 weeks following fat grafting into the left reconstructed breast; this was confined to the irradiated chest wall skin; the unirradiated LD flap skin paddle was not affected by this tissue reaction.

**Figure 3:**
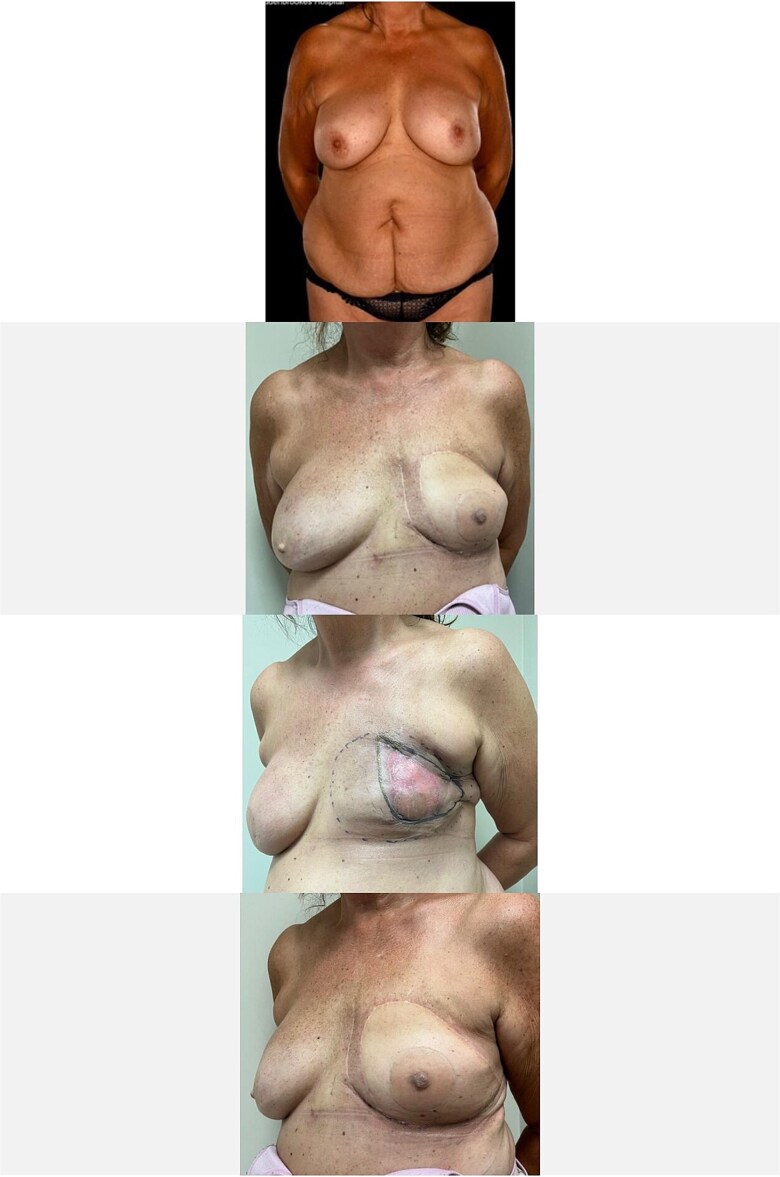
Final outcome following salvage reconstruction compared to the initial premastectomy pre-DIEP flap appearances, the pre-LD appearances. Patient has a left prosthetic nipple and a right reconstructed and tattooed nipple; there has been an improvement in the appearance of the index left breast; the right unirradiated DIEP flap-reconstructed breast had no changes.

Three weeks following autologous fat grafting (AFG), she developed tender, indurated erythematous nodules in the irradiated native skin raising concern for locoregional recurrence (LRR) (Fig. 2). Ultrasound and CT scanning excluded recurrence, revealing evolving fat necrosis. Core biopsies showed sinus tracts with active inflammation, abscess formation, and foreign body giant cell reaction. Debridement, washout, and advancement of the LD flap improved wound healing. Subsequent AFG addressed residual volume loss and asymmetry. A Becker-35™ expander was placed but subsequently removed as per the patient’s request. Two further AFG cycles followed; the patient is satisfied with the outcome (Fig. 3).

## Discussion

AFG is oncologically safe, with no increased LRR after breast-conserving surgery or mastectomy regardless of radiotherapy [7], [8] However, fat necrosis and palpable nodularity complicate cancer surveillance, particularly in the irradiated breast [9]. We describe a florid nodular reaction in irradiated native skin 3 weeks post-AFG, earlier than previously reported, which typically evolves over months [9]. To our knowledge, this acute presentation in irradiated native skin is unreported and has direct implications for cancer surveillance and patient counselling.

### A diagnostic mimic of recurrence

The nodules arose in the irradiated native mastectomy skin envelope, a recognized site of cutaneous spread in invasive lobular carcinoma, a subtype prone to occult recurrence [10]. The heavily modified tissue background compounded uncertainty; the short postgrafting interval was the only feature favouring a benign cause. Any new indurated or erythematous lesion arising in irradiated native skin after lipofilling should be regarded as indeterminate until proven otherwise, regardless of timing.

### Proposed mechanism

The reaction likely reflects a maladaptive response to injected fat within a radiation-damaged microenvironment. Radiotherapy produces obliterative endarteritis and chronic hypoperfusion, compromising revascularization and increasing the volume of non-viable fat available as a pro-inflammatory stimulus. Radiation-induced lymphatic disruption impairs the clearance of cellular debris, sustaining the foreign body response seen on core biopsy [11]. Sustained upregulation of pro-inflammatory cytokines may convert a self-limiting response into a clinically florid one. A scarred recipient bed and a large single-stage volume (270 ml) likely compounded these effects [12].

The tissue response to fat grafting into an irradiated bed exists on a spectrum, determined by radiation dose, radiotherapy (RT) interval, recipient-bed vascularity, graft volume, and host immune factors [13]. Positioning each patient on this spectrum should inform preoperative planning [13].

### Implications for technique and counselling

Three potential practical implications follow from this case. First, graft volume per session should be reduced and the reconstruction staged across more sessions when the recipient bed is irradiated and additionally compromised by previous fat necrosis, flap salvage, or extensive subcutaneous scarring. Smaller per-session volumes reduce the absolute mass of non-viable graft, lowering the inflammatory stimulus to which the radiation-damaged microenvironment can respond. Second, inter-session intervals should be lengthened to permit angiogenic remodelling of the recipient bed before further graft is delivered; the conventional 3-month minimum may be insufficient in this scenario. Third, preferential placement of graft into better-vascularized planes, with limited deposition into the most heavily irradiated native skin, is a reasonable refinement.

Preoperative counselling should explicitly address the possibility of a benign inflammatory reaction mimicking LRR in the weeks following AFG. Forewarning attenuates the psychological impact of the diagnostic pathway, supports the patient’s engagement with imaging and biopsy when these are required, and reduces the risk of misattribution by the patient or by clinicians unfamiliar with this scenario.

### Limitations

A single case cannot establish causation, quantify risk, or identify susceptible patients. The granulomatous foreign body reaction was not a radiation-specific mechanism; a scarred recipient bed, a large (270 ml) single-stage graft volume, and Crohn’s disease may have contributed. Larger, standardized studies are needed to define the incidence, risk factors, and preventive techniques.

## Conclusion

This case reports a clinically significant and previously unrecognized complication: a florid nodular inflammatory reaction after fat grafting into irradiated native breast skin that can mimic LRR. Any postlipofilling nodular/ indurated lesion in irradiated skin should be considered indeterminate until proven otherwise. A staged, low-volume approach and preoperative counselling about possible inflammatory mimics are advisable to mitigate unnecessary diagnostic anxiety.
